# Successful Case of Lithotomy and the Removal of Three Calculi, in a Boy Three Years Old

**Published:** 1845-01

**Authors:** Rufus L. Haywood

**Affiliations:** Tuscaloosa, Alabama


*Successful Case of Lithotomy and the removal of three Calculi, in a boy three years old.* By Rufus Haywood, M. D., of Tuscaloosa, Alabama.

Communicated in a letter to Professor Pancoast.

In the month of April last, a boy three years old, the son of Mr. Walker, of Livingston, was brought to me with stone in the bladder. He had shown symptoms of it from the time he was six weeks old. These became more and more aggravated until he was two years old. At this age the pain became excessive, and the straining to pass his urine caused the rectum to protrude at each effort about two inches, showing a highly injected condition of the mucous coat. This symptom continued to the time of the operation. During the previous summer and winter he had attacks of intermittent fever. When he came to Tuscaloosa, he had tertian intermittent.

In two or three weeks he was relieved of the fever, and his general health was much improved. His system being now in as good condition as, from his previous history, I could reasonably expect to get it, I concluded that farther delay would be unnecessary. I therefore performed the operation, and extracted three calculi, the aggregate weight of which would probably have been two ounces.

The lateral operation was the one adopted; and I never had a patient to do better after an operation for the space of six or seven days. The case, at this time, became complicated with diarrhœa, (which was then prevailing as an epidemic,) which lasted twelve or fifteen days, embarrassing the treatment and retarding the cure.

The diarrhœa was at length arrested, and his health in a short time entirely restored.

The protrusion of the rectum, which was to him a source of as great annoyance as the calculi themselves, disappeared from the time of the operation, and he has had no symptoms of it since. The operation succeeded perfectly.

*Tuscaloosa, December* 11, 1844.

The following analysis of the calculi so successfully removed by Dr. Haywood, in this very interesting case, was made by Professor Brumley, of the University of Alabama.

Uni, May 18, 1844.

*My Dear Sir,*—As I supposed your treatment of your patient might require an immediate examination of the calculus which you gave me this morning, I began the analysis of it soon after you left my laboratory.

In its size, oval form, yellowish brown colour externally, and yellowish white internally, laminated structure, smooth and hard, yet blistered surface, and comparative brittleness, it appeared to me to agree with the descriptions given by chemists of either the uric acid or the phosphate of lime calculus.

In water it is insoluble, but emits a few bubbles of air, softens slightly, loses colour, swells, and yields a small quantity of mucus, &c.

In ammonia, cold or hot, it whitens, but does not dissolve. It is insoluble in hot diluted muriatic acid. Nitric acid dissolved it readily with violent effervescence, owing to the formation and escape of carbonic acid. The solution, nearly colourless, being evaporated on a plate of glass, yielded a brownish matter, the edges of which were coloured very distinctly with the purple tinge of purpurate of ammonia, now called muroxid. A piece of it was heated on charcoal. It became black, did not fuse, gradually evaporated, or was consumed, leaving a mere trace of earthy matter, the nature of which I did not examine, as I was sure it was not phosphate of lime. Another piece, large as a pea, was then heated to redness in a long test tube of small diameter. It was not fused, became black, and sublimed, except a small quantity of charcoal. On the sides of the tube a white solid was deposited. Another portion was heated in a solution of pure potassa, and was readily dissolved. From the solution, dilute muriatic acid threw down a white precipitate, which was found to be *uric acid.* I could not discover any odour of ammonia.

I am, therefore, satisfied, that the specimen you gave me is a remarkably distinct example of the uric acid calculus. I began its examination with the belief, that it would prove to be the phosphate of lime. I was mistaken. The results of all the experiments were so distinct, that I wish you had been with me.

Yours truly,

R. T. BRUMLEY.

